# The pedfix technique: a new minimally invasive method of peritoneal dialysis catheter insertion with secure fixation to the abdominal wall—a preliminary experience

**DOI:** 10.1007/s00383-026-06437-z

**Published:** 2026-05-04

**Authors:** Salvatore Cascio, Mariateresa Cascio, Ibraheem Abdelraheem

**Affiliations:** 1 Department of Paediatric Urology, Children’s Health Ireland at Temple Street, Dublin, Ireland; 2 School of Medicine, University College Dublin, Dublin, Ireland; 3 St Vincent Hospital, Dublin, Ireland

**Keywords:** Peritoneal dialysis catheter, Blockage, Malposition, Laparoscopy, Tenckhoff catheter, End stage renal disease, Catheter fixation

## Abstract

**Purpose:**

Peritoneal Dialysis (PD) is the most common modality of renal replacement therapy in children with acute kidney injury (AKI). Various open and laparoscopic techniques for the placement of peritoneal dialysis (PD) catheter, with or without intra-abdominal fixation, have been described. The incidence of catheter migration after PD is 0–19%. We present a new, modified technique which minimizes the risk of catheter migration and blockage.

**Materials and methods:**

For 18 years a laparoscopic assisted PD catheter insertion has been used by the first author with a single 5 mm Port at the umbilicus using an inverted J incision and a Seldinger technique, with either a 14 or 16 Fr introducer kit. In October 2024, the technique was modified to include fixation of the catheter to the lower anterior abdominal wall using an Endoclose device (Autosuture, Covidien).

**Results:**

Six patients- 4 boys, mean age at surgery 7.8 years- underwent PD catheter insertion using the modified technique. All patients were in End Stage Kidney Disease due to posterior urethral valves and renal dysplasia (2), nephronophthisis (1) renal dysplasia (1), focal segmental glomerulosclerosis (1) multi-cystic dysplastic kidney on one side and dysplasia on the contralateral side (1). All six cases underwent successful placement of a PD catheter. Four patients had no post-operative complications, one patient developed an incisional hernia at the Hasson port, while one patient required laparoscopic repositioning of the PD catheter and fixation to the abdominal cavity with a non-absorbable suture (2 − 0 Ethibond) at a mean follow up of 10.4 months.

**Conclusion:**

The Pedfix technique provides excellent cosmetic results, optimal pelvic visualization and fixation of the PD catheter to the lower anterior abdominal wall which minimizes the risk of catheter migration and blockage. These encouraging results need to be confirmed by a prospective study with longer follow up.

## Introduction

Peritoneal Dialysis (PD) is the most common modality of renal replacement therapy in children with acute kidney injury (AKI) [1]. For the placement of Tenckhoff catheter, multiple techniques have been described over the years including percutaneous, open surgical and laparoscopic [2]. The minimally invasive approach for PD catheter insertion in children has been described since the late 1990s with modifications over the years using one, two or three ports [3, 4]. It offers several advantages including smaller incisions, reduced postoperative pain, shorter hospital stays and faster recovery that may contribute to the lower incidence of infectious complications and subsequent peritonitis [5–7]. In addition, laparoscopic techniques using a standardized “3 in 1” approach enable excellent visualization of the peritoneal cavity, allowing for precise positioning of the PD catheter under direct vision in the pelvis, omentectomy and closure of patent processus vaginalis [8]. The laparoscopic approach also enables the exploration of a blocked PD catheter and facilitates management of unexpected anatomical variations such as congenital bands, removal of entrapped omentum or fibrinous debris around the catheter, lysis of adhesions or repositioning the catheter into the retro vesical or Douglas pouch [9].

PD catheter failure is commonly reported with rates ranging from 30% at 2-month post-placement, to 20% at 12-months post-placement [10, 11] with an overall 19% of patients staying on PD until kidney transplant [9]. Of the failed PD catheters requiring replacement over a third (36%) are due to leakage and another third (32%) to either infection or migration [9]. In order to reduce the incidence of catheter migration and blockage various modifications have been applied to the laparoscopic approach to secure the PD catheter to the abdominal wall. Laparoscopic Endoclose Fixation (LEF) has recently been described in adults using a three ports approach. It has shown to be a simple and safe method of reducing catheter malposition and blockage [12]. We present the PEDFIX method which combines the single port laparoscopic percutaneous technique of PD catheter insertion with the Endoclose Fixation which minimises the risk of catheter migration and blockage.

## Materials and methods

### Surgical technique

**Fig. 1:**
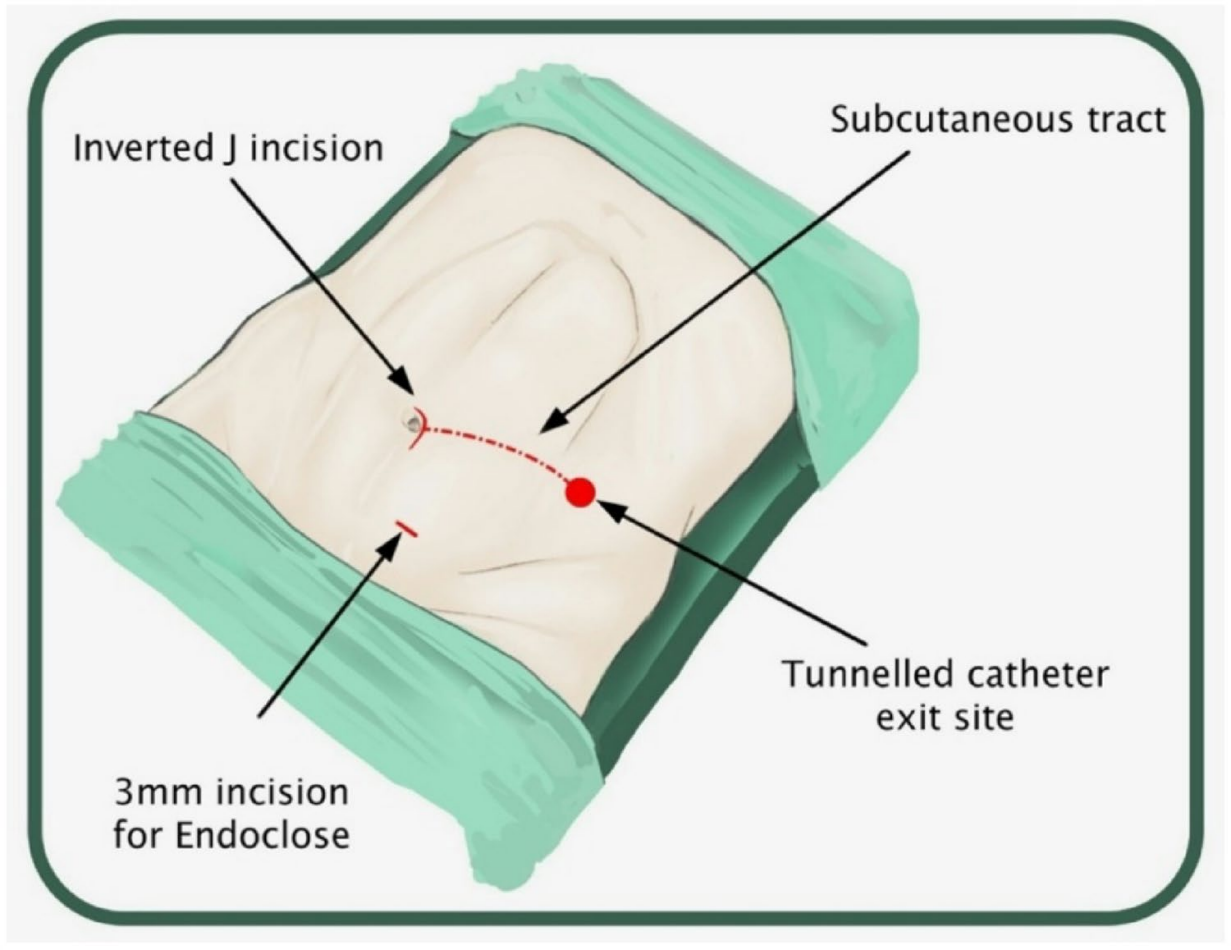
Supra-umbilical “inverted J” incision on the left side of the umbilicus, subcutaneous track + exit site of PD catheter and 3 mm incision for Endoclose

**Fig. 2:**
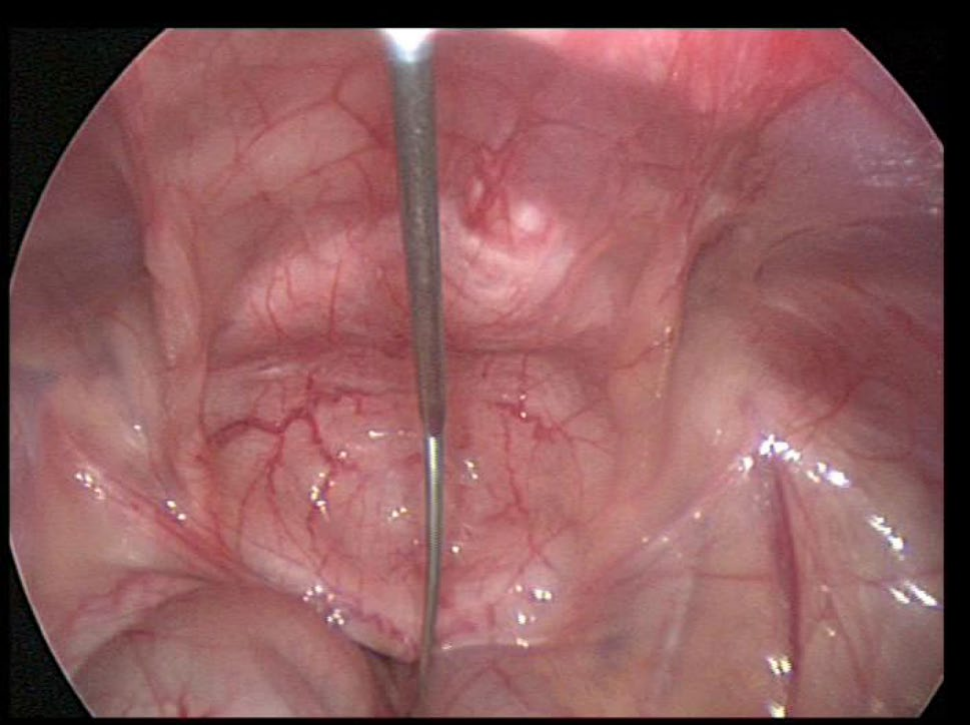
The double ended guidewire is advanced through the needle into the pelvis

**Fig. 3:**
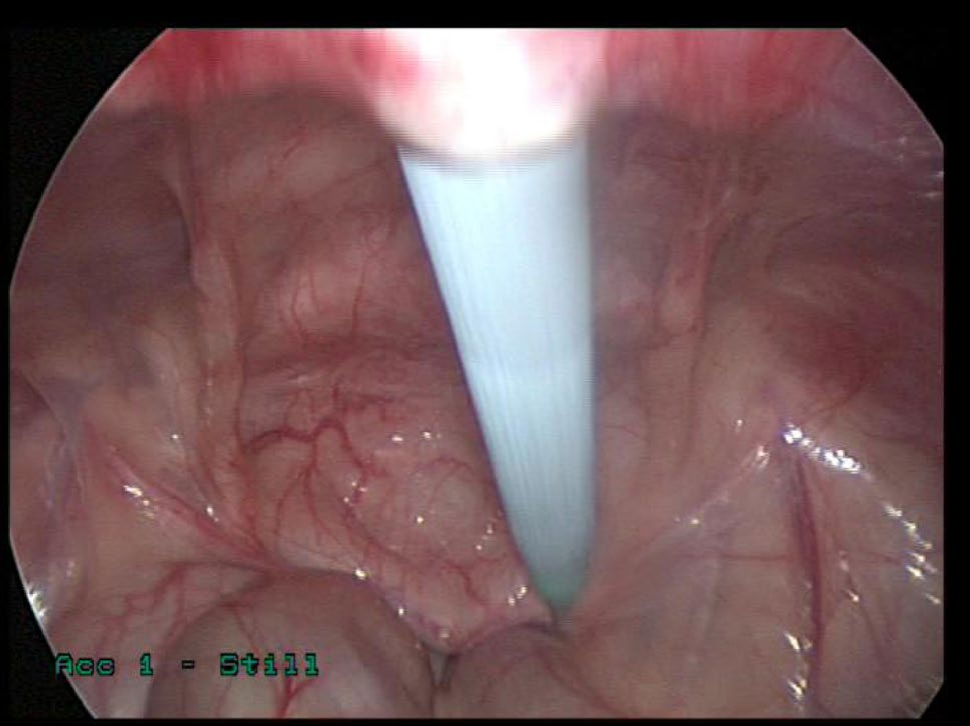
A 14 Fr peel away sheath and dilator inserted over the wire

**Fig. 4:**
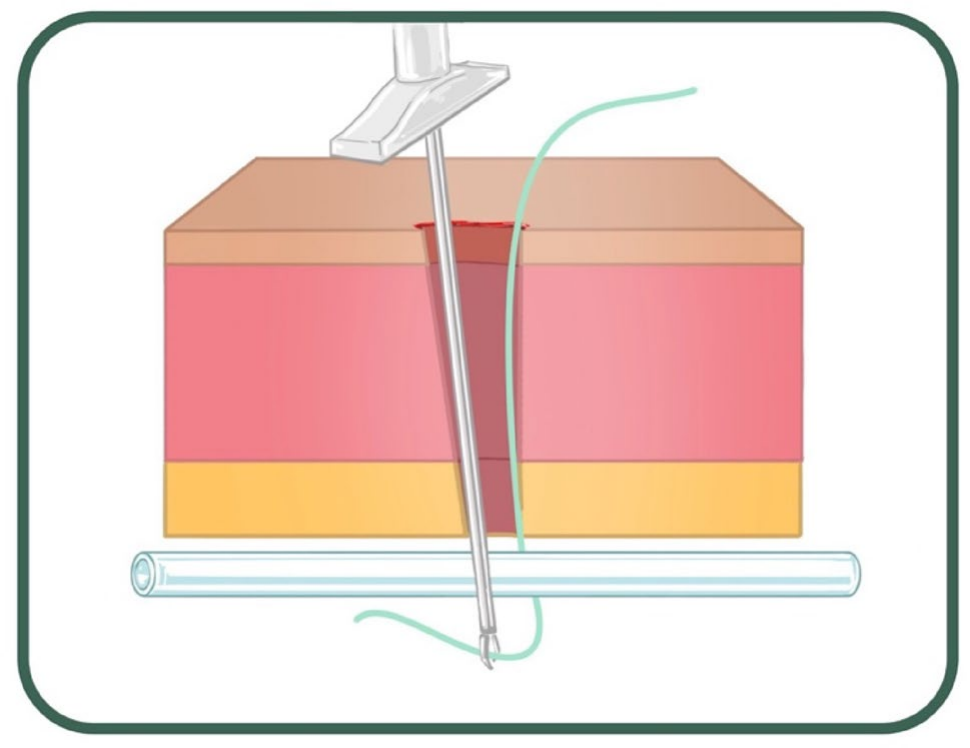
Endoclose reinserted on the opposite site of the sheath, to hold the suture and pull it outside the abdominal cavity

**Fig. 5:**
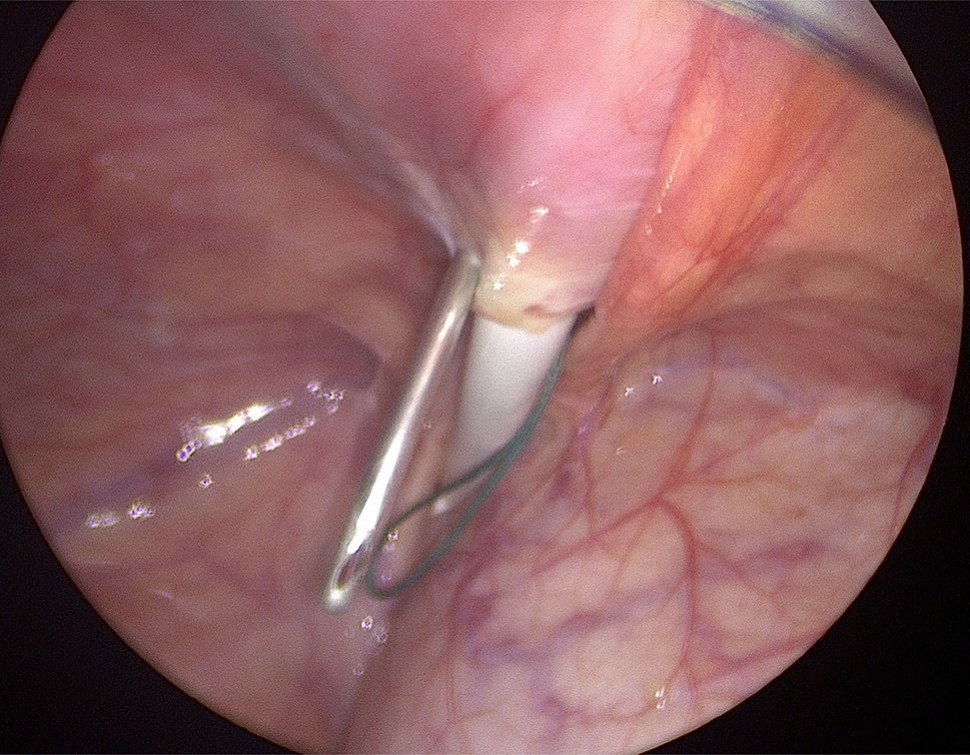
Non absorbable suture around the peel away sheath

**Fig. 6:**
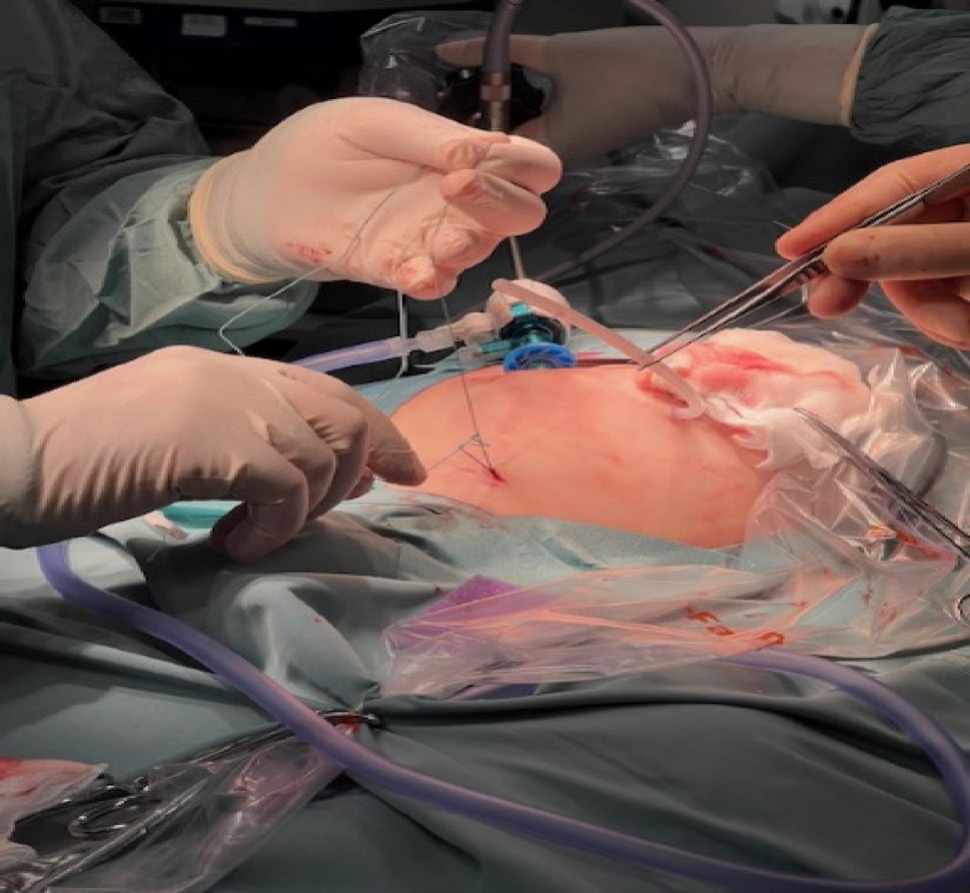
Non absorbable suture tied extra-corporeally around the PD catheter,

**Fig. 7:**
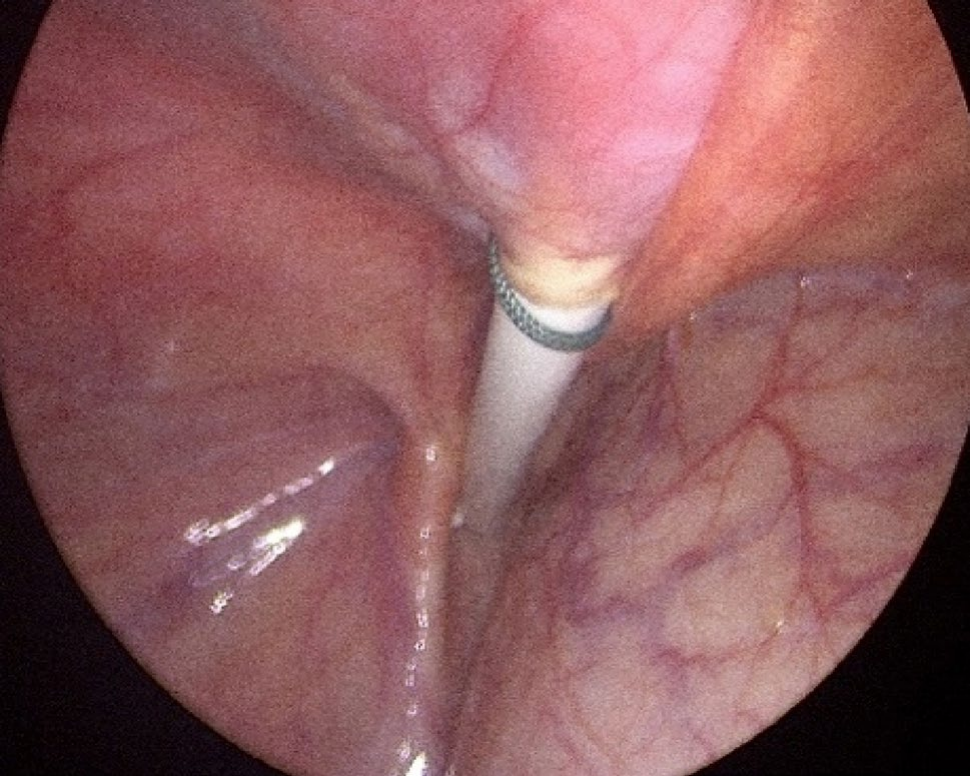
PD catheter secured to the anterior abdominal wall

A supra-umbilical “inverted J” incision is made on the left side of the umbilicus (Fig. 1). Monopolar diathermy is used to divide the subcutaneous tissue and to expose the anterior rectus sheath below the umbilicus. With the index finger, by blunt dissection, a space for the cuff is created between the anterior rectus sheath and the subcutaneous tissue. The peritoneal cavity is entered with a standard Hasson technique above the umbilicus. It is essential to remove as much omentum as possible to minimize catheter blockage. The omentum is grasped with forceps and delivered outside the abdominal cavity, tied with ligature and excised. The abdomen is insufflated with carbon dioxide to a pressure of 10–15 mmHg according to the age of the child. A long oblique tract in the abdominal wall extending from the infraumbilical space towards the pelvis, through the rectus muscle, is created with a large needle under laparoscopic vision (the needle at 30-degree angle with the abdominal wall). This allows the catheter to be sited in the pelvis and the obliquity of the track to prevent leakage. The double ended guidewire is advanced through the needle into the pelvis (Fig. 2). The needle is removed and, either a 14 Fr or 16 Fr peel away sheath and dilator, are inserted over the wire (Fig. 3). The dilator is removed and a double cuffed peritoneal dialysis catheter is fed through the sheath toward the pelvis, behind the bladder. A stab incision is made with an 11 Blade to the left in the lower abdominal wall and an Endoclose (Covidien) and an Ethibond 2 − 0 (Ethicon, US) suture attached to it, is introduced into the abdominal cavity around the sheath. Once the suture is inside the abdominal cavity, the Endoclose is pulled out and reinserted on the opposite site of the sheath, to hold the Ethibond suture and pull it outside the abdominal cavity (Figs. 4 and 5). Once the catheter is inside the pelvis, the sheath is peeled away leaving the peritoneal dialysis catheter in the pelvis and the Ethibond suture is tied extra-corporeally (Fig. 6) around the PD catheter, securing the catheter to the anterior abdominal wall (Fig. 7). A 1 cm incision is made lateral to the rectus muscle on the left. A long-curved mosquito forceps is introduced into the incision to create a subcutaneous tunnel towards the umbilicus (Fig. 1). At the umbilicus the catheter is grasped with the curved mosquito forceps and pulled towards the exit site. It is important that the cuff is at least 2 cm away from the exit site. The abdomen is deflated, the Hasson port closed with a 2 − 0 Vicryl purse string, subcutaneous approximated and skin closed with topical skin adhesive.

## Materials and methods

Six patients, 4 boys/2 girls, mean age at operation 7.8 years, underwent placement of a PD catheter using the PEDFIX technique during the period October 24-Dec 25. All patients were in End Stage Kidney Disease due to posterior urethral valves and renal dysplasia (2), nephronophthisis (1) renal dysplasia (1), focal segmental glomerulosclerosis (1) multi-cystic dysplastic kidney on one side and dysplasia on the contralateral side (1). For three patients it was the first PD catheter insertion, for two patients it was the second insertion while for the last patient it was the fourth placement. Four patients had no post-operative complications. One patient developed an incisional hernia at the Hasson port. In the fourth patient of the series, the PD catheter became blocked and was noted to have moved to the left upper abdominal quadrant, requiring a laparoscopic replacing of the catheter and securing to the anterior abdominal wall with a *non-absorbable suture* (2 − 0 Ethibond, Ethicon, US). Since then we have changed our technique and we always use a non-absorbable suture with no further complications at a mean follow up of 10.4 months.

## Discussion

The placement of Peritoneal dialysis (PD) was first described by Tenckhoff in 1968 [13]. Over the years ambulatory PD has become the preferred method of renal replacement therapy in children with AKI and End Stage Kidney Disease (ESKD) because it is cost-effective, has a lower incidence of serious complications, improves patient nutrition and independence [2]. Different methods for placement of PD catheters are available which include the traditional open surgical technique, the percutaneous Seldinger technique and the laparoscopic approach [2]. In 1997 Brownlee and Elkhairi described the placement of a PD catheter in 10 patients under laparoscopic view [3]. Two years later, Lessin et al. presented a laparoscopic placement and suturing of a PD catheter through a single supraumbilical incision. The authors described a gasless technique and used a needle holder placed alongside the lens and the Tenckhoff catheter to secure the catheter to the medial umbilical ligament [4]. The published literature would support the use of the laparoscopic approach versus the open technique and fixation of the catheter to the anterior abdominal wall. A recent systematic review and meta-analysis comparing laparoscopic versus open placement of PD catheters in paediatric patients identified a total of nine studies comprising 659 children. The study demonstrated that with the laparoscopic approach the odds of requiring reoperation and of developing peritonitis were reduced by more than 70% and 80% respectively [14]. The lower risks of re-operation and peritonitis with the laparoscopic approach can be easily explained by the smaller incisions, shorter hospital stay and better visualization of the peritoneal cavity which allows a precise localization of the catheter in the pelvis. Catheter malposition and blockage is a common complication following PD catheter insertion with the incidence ranging from 0 to 19% [12, 15]. Catheter blockage and failure is significantly higher when omentectomy is not performed at the time of catheter placement [9–11] and when the catheter has not been sutured to the pelvis, especially in children in whom the pelvis is shallow and the catheter tip can be easily dislodged [16–18]. To reduce the incidence of catheter migration and blockage different modifications have been applied to the laparoscopic approach to secure the PD catheter to the abdominal wall. Krezalek described a rectus sheath tunnelling and selective omentopexy while Shen et al. used a technique of suture-passer hernia forceps, reporting 0% migration in 39 patients with a follow-up period between 6 and 42 months [15, 19]. Gunes et al. described a technique of preperitoneal tunnelling and extracorporeal pelvic suture fixation resulting in an 8.5% rate of catheter dysfunction and migration [20]. Recently Rouse et al. presented the results of 145 adults who underwent placement of PD using a three ports technique with fixation of the PD catheter to the midline of the abdominal wall using 2/0 Prolene suture with an EndoClose device [12]. PD catheter malposition was identified in 12/63 (19.05%) of the patients in the non-suturing group versus 6/82 (7.32%) of the suturing group. The techniques described in our study combine, for the first time, the use of a single port laparoscopic percutaneous approach with fixation of the catheter to the anterior abdominal wall without laparoscopic instruments, using an Endoclose device. This is a preliminary report including only six patients and more prospective studies are needed to confirm our encouraging results. The technique described offers the advantages of a minimally invasive approach, better cosmesis, shorter hospital stay, reduced post operative pain and better visualization of the pelvis, with securing of the catheter to the abdominal wall which reduces dislodgement and obstruction of the catheter. We acknowledge that in one of our patients the catheter migrated to the left upper quadrant due to a technical mistake of having used an absorbable suture. Since then, we have switched to a non-absorbable suture (2 − 0 Ethibond) to secure the catheter to the anterior abdominal wall, with no further dislodgments or obstruction of the catheter.

## Conclusions

The Pedfix technique gives excellent cosmetic results combining easy access to the abdominal cavity for an omentectomy, an optimal view of the pelvis for precise localization of the PD catheter with a fixation of the catheter to the lower anterior abdominal wall, which minimizes the risk of catheter migration and blockage. Further prospective studies are recommended to confirm these encouraging results.

## Data Availability

No datasets were generated or analysed during the current study.
